# Barriers and Facilitators to the Implementation of the Early-Onset Sepsis Calculator: A Multicenter Survey Study

**DOI:** 10.3390/children10101682

**Published:** 2023-10-12

**Authors:** Liesanne E. J. van Veen, Bo M. van der Weijden, Leti van Bodegom-Vos, Jeroen Hol, Douwe H. Visser, Niek B. Achten, Frans B. Plötz

**Affiliations:** 1 Department of Paediatrics, Tergooi MC, Laan van Tergooi 2, 1212 VG Hilversum, The Netherlands; l.vanveen@franciscus.nl (L.E.J.v.V.); b.m.vanderweijden@amsterdamumc.nl (B.M.v.d.W.); 2 Department of Paediatrics, Franciscus Gasthuis en Vlietland, Kleiweg 500, 3045 PM Rotterdam, The Netherlands; 3 Department of Paediatrics, Erasmus MC, Sophia Children’s Hospital, Wytemaweg 80, 3015 CN Rotterdam, The Netherlands; n.achten@erasmusmc.nl; 4 Amsterdam UMC, Department of Paediatrics and Amsterdam Reproduction & Development Research Institute, Location University of Amsterdam, Emma Children’s Hospital, Meibergdreef 9, 1105 AZ Amsterdam, The Netherlands; d.h.visser@amsterdamumc.nl; 5 Department of Biomedical Data Sciences, Medical Decision Making, Leiden University Medical Center, Albinusdreef 2, 2333 ZA Leiden, The Netherlands; l.van_bodegom-vos@lumc.nl; 6 Department of Paediatrics, Noord West Ziekenhuis, Wilhelminalaan 12, 1815 JD Alkmaar, The Netherlands; j.hol@nwz.nl; 7 Amsterdam UMC, Department of Neonatology, Emma Children’s Hospital, Meibergdreef 9, 1105 AZ Amsterdam, The Netherlands

**Keywords:** barriers, early-onset sepsis, facilitators, implementation, neonatal early-onset sepsis calculator, neonates, stakeholders

## Abstract

Prior studies demonstrated the neonatal early-onset sepsis (EOS) calculator’s potential in drastically reducing antibiotic prescriptions, and its international adoption is increasing rapidly. To optimize the EOS calculator’s impact, successful implementation is crucial. This study aimed to identify key barriers and facilitators to inform an implementation strategy. A multicenter cross-sectional survey was carried out among physicians, residents, nurses and clinical obstetricians of thirteen Dutch hospitals. Survey development was prepared through a literature search and stakeholder interviews. Data collection and analysis were based on the Consolidated Framework for Implementation Research (CFIR). A total of 465 stakeholders completed the survey. The main barriers concerned the expectance of the department’s capacity problems and the issues with maternal information transfer between departments. Facilitators concerned multiple relative advantages of the EOS calculator, including stakeholder education, EOS calculator integration in the electronic health record and existing positive expectations about the safety and effectivity of the calculator. Based on these findings, tailored implementation interventions can be developed, such as identifying early adopters and champions, conducting educational meetings tailored to the target group, creating ready-to-use educational materials, integrating the EOS calculator into electronic health records, creating a culture of collective responsibility among departments and collecting data to evaluate implementation success and innovation results.

## 1. Introduction

Use of the Early-Onset Sepsis (EOS) Calculator, a risk-assessment tool helping physicians narrow antibiotic use in neonates at risk for EOS, is quickly spreading internationally. The calculator was developed by the Kaiser Permanente Research division and is based on a multivariate risk prediction model, combining both maternal intrapartum factors and objective neonatal clinical findings to estimate individual EOS risk and subsequently give policy recommendations [1,2,3]. Studies comparing the EOS calculator to conventional management strategies showed a significant decrease in neonatal empiric antibiotic use [4,5,6,7]. A number of Dutch hospitals already use the calculator in the study context of a multicenter cluster randomized controlled trial [8]. Similar to its inclusion in the latest National Institute for Health and Care Excellence (NICE) guidelines [9], the tool is likely to be included in the upcoming revision of the Dutch national guidelines, as well as in international guidelines, and will be implemented in clinical practice.

However, successful implementation of a revised guideline is not self-evident, as the translation of novel evidence into daily clinical practice is found to often be suboptimal [10]. Three large systematic reviews on guideline adherence in different healthcare settings showed adherence rates ranging from 0 to 98%, with mean rates around 50–70% [11,12,13]. A study investigating adherence to the current Dutch guidelines on EOS management reported only 42.5% adherence to recommendations regarding the start of antibiotic treatment [14]. Though no data are available yet on the EOS calculator adherence in daily clinical practice, few effectiveness and safety studies did mention some concerns regarding EOS calculator use, including safety concerns, incorrect use and practical concerns, which may contribute to low adherence rates [15,16,17,18]. Since low guideline adherence leads to variations in daily clinical practice, a waste of resources and suboptimal patient outcomes, research should not end with evidence for a guideline or tool but continue with studies on how to achieve successful implementation [10,19,20]. Prior to implementing an innovation, gaining insight into the factors that may hinder or promote its adoption is an essential step in the preparation process.

The primary objective of this study is to identify the key barriers and facilitators perceived by stakeholders of EOS calculator implementation. By understanding these factors, this study seeks to inform a comprehensive implementation strategy that will optimize the successful integration and utilization of the EOS calculator in practice.

## 2. Materials and Methods

### 2.1. Study Design and Participants

A multicenter cross-sectional online survey was conducted among stakeholders of EOS calculator implementation in thirteen Dutch hospitals. For selecting hospitals, purposive sampling was used in order to reflect different types of neonatal care in the Netherlands and ensure representation of all groups of stakeholders. Among included hospitals, the highest level of neonatal care was the Neonatal Intensive Care Unit (NICU) in 2 hospitals, High Care in 7 hospitals and Medium Care in 4 hospitals. The NICUs are categorized as tertiary care facilities, offering the most advanced level of care for critically ill neonates and severely preterm infants (<30 weeks of gestation). High-Care departments are situated at regular pediatric departments and provide care for ill neonates and preterm infants >30 weeks of gestation, facilitating continuous monitoring and respiratory support. Medium-Care departments are also situated at regular pediatric departments and provide simple medical care for neonates >32 weeks of gestation. In some cases, the care provided in the Medium-Care department may be extended to the maternity ward, contingent on local agreements.

To identify relevant stakeholder groups, Dutch workflows of managing neonates at risk for EOS were explicated. In case of EOS calculator use, physicians of a neonatology ward examine neonates at risk for EOS and fill in the EOS calculator as they are responsible for neonatal policy. Physicians of the obstetrics ward play an important role in sampling and sharing information about maternal factors needed for the EOS calculator. Nurses of both the neonatology and obstetrics wards have to execute some of the calculators’ practical recommendations, such as measuring neonatal vital signs. Based on this workflow, four main groups of stakeholders were identified: (1) physicians of the neonatology ward (PN) (pediatricians, neonatologists, pediatric residents and physician assistants); (2) physicians of the obstetrics ward (PO) (gynecologists, perinatologists, clinical obstetricians and gynecologic residents); (3) nurses of the obstetrics ward (NO) (maternity nurses and obstetric nurses); and (4) nurses of the neonatology ward (NN) (NICU nurses, neonatal nurses and pediatric nurses).

### 2.2. Survey Development

**Figure 1:**
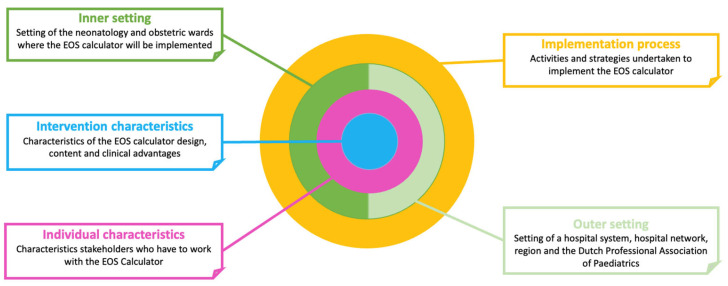
Graphic overview of the five domains of the CFIR, focused on the setting of this study.

The Consolidated Framework for Implementation Research (CFIR) was used to structure survey development and analysis. The CFIR is a frequently used determinant framework and combines different theories and existing models into a list of 5 domains and 39 constructs of implementation success in healthcare settings [21]. The five domains are (1) intervention characteristics, (2) outer setting, (3) inner setting, (4) characteristics of individuals and (5) implementation process (Figure 1). Steps of survey development were informed by the guide of Burns et al. on designing and conducting self-administered surveys of clinicians [22]. The CROSS checklist (Consensus-Based Checklist for Reporting of Survey Studies) was used as a reporting checklist [23].

#### 2.2.1. Survey Preparation

To explore potential barriers and facilitators of EOS calculator implementation and thereby identify relevant survey items, a preparatory literature search in Pubmed was combined with 12 exploratory semi-structured stakeholder interviews. Facilitators and barriers identified in literature an during the interviews were analyzed and summarized in a point-by-point list [16,17,18,24,25,26,27,28,29,30,31,32,33,34,35,36,37,38,39,40,41,42]. Subsequently, all factors were allocated to one of the 39 CFIR constructs using the CFIR codebook [21,43]. A more detailed description of both the methods and results of the survey preparation can be found in Supplementary Materials.

#### 2.2.2. Survey Item Generation

Item generation was based on barriers and facilitators identified in the literature and during the interviews. The list of generated items was reviewed by all authors. Items rated less relevant were individually marked and collectively reconsidered to reduce the number of items. To avoid non-response, a short and easy-to-understand survey design was pursued. All questions were formulated simply, as specific as possible and mostly in statement format. Questions focusing on more than one construct were avoided. Response format was predominantly a Likert-type 5-point scale, besides some multiple-choice options, yes/no answers and textboxes to clarify initial statements. For most questions, a ‘neutral’ or ‘no opinion’ answer was available. An implementation expert (author LBV) was consulted to review format of questions, answer options and questioning order. Face validity was assessed by researchers of the team as well as sample participants. The survey was pilot-tested in a small group of 8 persons reflecting the study sample (1 pediatrician, 2 pediatric residents, 1 gynecologist, 2 clinical obstetrician and 2 nurses). After testing, small changes were made to improve clarity and readability of questions.

#### 2.2.3. Final Survey

The final survey contained questions regarding all five domains of the CFIR. The first and main part was a general section, containing 18 questions for all respondents. The second part was a discipline-specific section, containing 4–7 questions per stakeholder subgroup. The third part was only applicable for participants who indicated to currently work with the EOS calculator and contained 3–4 questions per subgroup. The complete survey can be found in Table S2.

#### 2.2.4. Survey Dissemination

Survey dissemination: In August 2022, the online survey was carried out among all stakeholders currently employed in the included hospitals. The survey was set out in Castor Electronic Data Capture System where a survey link was created. The survey and cover letter were sent by secretariats of participating departments to individual email addresses of 2054 eligible stakeholders, including 387 PN, 442 PO, 630 NO and 586 NN. To improve response rate, reminders were sent ten days and three weeks after the survey went live. The survey link was open for two months.

### 2.3. Data Analysis

Survey data were exported and analyzed using IBM SPSS Statistics 28.0. Only fully completed surveys were included for analysis. Likert-data were coded from 1 to 5, with 1 = totally disagree/very unimportant to 5 = totally agree/very important. Reversely formulated statements were recoded so that a higher score indicated participant’s stronger association with the topic. For data analysis and presentation, Likert-data were divided into 2 categories: irrelevant/neutral (I) = 1 + 2 + 3 and relevant (R) = 4 + 5. Relevant facilitators were defined as facilitators that were rated ‘R’ by >50% of respondents. Relevant barriers were defined as barriers that were rated ‘R’ by >10% of respondents, as barriers applicable to only small numbers of stakeholders may also have a large impact on implementation outcomes. For all closed questions, the frequency distribution of reported barriers and facilitators in the total group and, if applicable, sub-groups were calculated in percentages. If deemed relevant for practice, differences between subgroups were tested using the chi-square test of independence, followed by post hoc Bonferroni testing. *p*-values of <0.05 were considered statistically significant. Open-ended questions were analyzed through open coding. Abbreviations (PN, PO, NO, NN) were used to describe subgroups.

## 3. Results

### 3.1. Participants and Guidelines

**Figure 2:**
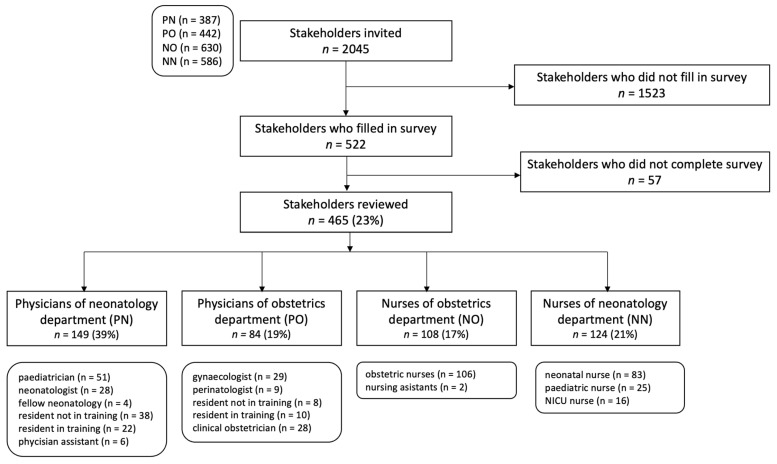
Study flow chart.

The survey was sent to 2054 eligible participants of 13 Dutch hospitals. The survey link was opened 1154 times, resulting in 522 survey responses, of which 465 (23%) were fully completed (Figure 2). The response rate was 39% in the PN group, 20% in the PO group, 17% in the NO group and 21% in the NN group. No information on unique visitors was available. Of the 465 respondents, 40.2% reported to use the current Dutch guidelines, 16.6% the EOS calculator, 9.9% a local protocol, 12.7% a combination of protocols and 20.6% reported unknown.

### 3.2. Survey Part 1: Reported Barriers and Facilitators by All Respondents

Relevant facilitators and barriers for the total group of respondents, allocated to the CFIR domain, are displayed in Table 1. Relevant barriers were found only in the inner setting domain and concerned capacity shortage and problems with maternal information transfer between the departments. Capacity shortage was significantly more reported by physicians and nurses of the obstetrics ward compared to those of the neonatology ward and was mainly expected at the maternity ward, both due to staff and room shortage (Figure S1). Problems with the handover of maternal information from the obstetric to neonatology department were significantly more reported by physicians of the neonatology ward, compared to the other three subgroups.

Relevant facilitators were found in all five domains of the CFIR, though the majority of facilitators concerned intervention characteristics, including the reduction in neonatal antibiotic prescriptions, reduction in neonatal side-effects, less mother–child separation, reduction in blood tests and net shorter hospital stays. Endorsement of the EOS calculator by the NVK (outer setting), integration of the EOS calculator in EHR systems (inner setting), the belief that the EOS calculator is safe and effective (individual characteristics), the provision of training on the EOS calculator and the provision of feedback on implementation results at the own department (process) were found to be other relevant facilitators. An additional question regarding education showed clear differences in preferences between subgroups (Figure S2). An instructional video about the EOS calculator was chosen by the majority of all stakeholders. However, education about scientific evidence was specifically rated important by physicians, whereas a clinical lesson about EOS was clearly more preferred among nurses.

### 3.3. Survey Part 2: Reported Barriers and Facilitators per Group

Relevant facilitators and barriers reported by subgroups, allocated to CFIR domain, are displayed in Table 2. A PN-specific barrier was the belief that maternity nurses are not adequately trained to measure neonatal vital signs. PN-specific facilitators concerned tension for change of the current NVK guidelines and the general feeling that too many antibiotics are currently prescribed. PO-specific barriers concerned the expectation that the EOS calculator will increase workload, more neonates from primary care will be admitted to the hospital and the belief that maternity nurses are not adequately trained to measure neonatal vital signs. No PO-specific facilitators were found. NO-specific barriers concerned feeling incompetent to measure neonatal heart and respiratory rate and not being informed in a timely manner about changes in physicians’ protocols. NO-specific facilitators concerned the availability of an EOS calculator smartphone app, clear communication by physicians about reasons for policy choices and availability of a local implementation team. An NN-specific barrier concerned nurses not being informed in a timely manner about changes in physicians’ protocols. NN-specific facilitators concerned clear communication by physicians about reasons for policy choices and the availability of a local implementation team.

### 3.4. Survey Part 3: Reported Barriers and Facilitators by EOS Calculator Users

Relevant facilitators and barriers reported by EOS calculator users, allocated to the CFIR domain, are displayed in Table 3. Facilitators for the total group of EOS calculator users concerned the feeling that the care for neonates is more uniform since the implementation of the EOS calculator and the thought that parents agree with EOS calculator recommendations. A PN-specific barrier concerned encountering some textual or substantive uncertainties when using the EOS calculator. A PN-specific facilitator concerned the experience that the EOS calculator is supportive in making clinical decisions.

## 4. Discussion

To our knowledge, this study described the first systematical pre-implementation evaluation of factors influencing implementation of the EOS calculator. Our primary focus centered on identifying the barriers and facilitators of EOS calculator implementation as perceived by stakeholders within Dutch hospitals. Analysis revealed the presence of facilitators and barriers across all five domains of the CFIR. The facilitators were generally applicable across diverse stakeholder groups, while the barriers exhibited a tendency to be more discipline-specific.

Two main organizational barriers to EOS calculator implementation emerged: insufficient capacity in neonatal care departments and challenges in transferring maternal information between departments. Notably, capacity concerns were more prominent among obstetric physicians and nurses, likely tied to issues primarily expected at the maternity ward. However, this expectation contrasts with research indicating shorter hospital stays for neonates when using the EOS calculator [41]. The discrepancy might be attributed to the anticipated shift of care from the neonatology ward to the maternity ward, accompanied by increased clinical examinations.

Since comprehensive maternal information is vital for utilizing the EOS calculator effectively, streamlining the process of obtaining and transferring these data is critical. Respondents in this study suggested implementing standardized consultation forms with smart text features and enabling autofill for the EOS calculator through integration into the EHR. Similar strategies, like checklists or fill-in-the-blank handovers, have proven successful in improving information transfer in other healthcare settings [44,45].

Moreover, this study identified a barrier among nurses in the obstetrics departments, indicating that a small but relevant group of them does not feel confident in measuring neonatal respiratory rate and heart rate. This lack of self-efficacy is crucial to take into account, as it plays a significant role in determining one’s motivation to perform tasks [46]. Additionally, both groups of physicians expressed concerns about the adequacy of training for maternity nurses in measuring neonatal vitals. These combined findings emphasize the importance of implementing an intervention targeting this specific issue.

Facilitators identified in this study were several relative advantages of the EOS calculator, such as less mother–child separation and net shorter hospital stays. However, it is important to underscore that the extent to which relative advantages foster promotion is significantly contingent upon their visibility in daily practice [47,48]. Visibility may be improved through stakeholder education, wherein emphasis is placed on elucidating advantages. As stakeholders in this study have reported diverse educational preferences, the adaptation of educational methods for the target group should be considered. However, the standalone impact of education possesses certain limitations and should therefore be combined with other strategies [49,50]. The significance of evaluation and feedback has been widely recognized in the literature [47,51,52]. Facilitating this process involves acquiring and disseminating objective data pertaining to implementation success [53]. Notably, a recent meta-analysis revealed that the efficacy of feedback is strongly contingent on various factors such as the methodology employed, the recipients of the feedback and the context in which it is provided, thereby offering implications for practical implementation. For instance, feedback aimed at directly aiding clinical behavior demonstrated the highest level of effectiveness [54].

Integration of the EOS calculator in the EHR was deemed facilitative by a significant majority of stakeholders. This observation aligns with prior research, which demonstrated that the inclusion of clinical decision tools, exemplified by the EOS calculator, within the EHR, enhances their integration into clinical workflows [15,42,55,56]. Conversely, the importance of a smartphone application was only reported by a few stakeholders, which is an interesting finding, given that the concurrent RCT relies entirely on an EOS calculator smartphone application as its methodological approach [8]. The forthcoming findings from this RCT hold the potential to offer further insights and guidance on this subject matter.

The vast majority of all respondents expected the EOS calculator to be safe and effective. The reported general expectation of safety and effectivity is considered a beneficial starting point for implementing the EOS calculator. Tension for change of the current Dutch guidelines was reported by a large majority of physicians of the neonatology ward, as was expected based on these group’s reported low adherence to the current NVK guidelines [14]. It should be noted, however, that tension for change was not clearly present in the other groups of stakeholders, possibly because of their more indirect roles with regard to antibiotic use. It is crucial to pay attention to these stakeholder groups as a sense of urgency and the need for change are essential factors for the successful implementation of any new guidelines [57,58].

### 4.1. Strengths and Limitations

A major asset of this study was the inclusion of different groups of stakeholders, resulting in a complete overview of factors influencing implementation. By selecting hospitals with different levels of neonatal care, optimal reflection of the target population was pursued. As all stakeholders employed in the participating centers were invited to take part in the survey, we aimed to avoid selection bias. The survey being conducted before starting large-scale implementation ensures that the implementation can be tackled properly from the beginning. Still, additional factors may come forward during actual implementation, emphasizing the importance of evaluation. The CFIR framework, recommended for implementation projects, was used as available during the study, providing a robust theoretical base [59,60,61]. Currently, a revised CFIR has been published, which should be considered for future implementation research [62].

We recognize that our study has certain limitations. Firstly, the response rate was relatively low, which introduces a significant risk of non-response bias, a common challenge in healthcare professionals’ surveys [63,64,65]. As our aim was to obtain as many perspectives as possible and avoid selection bias, we chose to invite every single stakeholder in the included hospitals. Though many responses were gathered, this approach did not necessarily result in optimal response rates. While acknowledging non-response bias risk, we assert that our findings remain practically valuable. The absolute number of nearly 500 responses across 13 hospitals of varying care levels is robust and allowed for a diverse sample that effectively captured stakeholder perspectives. Although there is a chance that this study did not capture certain barriers or facilitators that non-responders could have brought to light, the factors identified in our research hold undeniable significance for their incorporation into clinical practice. Secondly, a clear disparity in response rate was seen among physicians from the neonatology ward (39%) compared to other stakeholders (PO:20%, NO: 17%, NN: 21%). It is described that people with more interest in the topic are more likely to answer a survey. At the time of our study, the EOS calculator was best known by physicians of the neonatal ward, mainly through pediatric journal publications and congresses. For physicians of the obstetric ward and both groups of nurses, the topic was further away, which likely contributed to lower response rates in these groups. Besides the lack of familiarity, the lack of time and the large number of surveys nurses may have diminished the response rate [66]. In our pilot phase, length and readability of the survey were not found to be an issue. Furthermore, hospitals typically have employed a greater number of physicians of the obstetrics ward and nurses, leading to a higher volume of surveys distributed to them compared to physicians in the neonatology ward. As the core importance of this topic rests with physicians of the neonatology ward, there might have been a relatively uneven distribution. Thirdly, potential bias was created by dichotomizing data for analysis into ‘relevant’ and ‘irrelevant’, ignoring the nuance of the ‘neutral’ option. However, it was well considered to do this, as this study’s aim was to identify genuinely relevant barriers and facilitators, which were not reflected by the ‘neutral’ option.

### 4.2. Clinical Implications

Our study provides a blueprint and benchmark for countries and networks looking to successfully implement the EOS calculator. Moreover, it may serve as an example for implementing other diagnostic score strategies aiming to narrow antibiotic use, such as serial clinical examination. Findings of this study may inform and facilitate an implementation strategy for the EOS calculator specifically (see Table 4), taking into account that additional strategies are needed when other factors come to light. Sufficient information supply is essential to both stimulate facilitators and tackle barriers. To show relative advantages of the EOS calculator and foster tension for change among stakeholders, local discussion and educational meetings should be conducted. Early adopters and local champions should be identified, so that they can be stimulated to motivate and educate colleagues. Education should consist of both ready-to-use materials, such as an instructional video, as well as educational meetings tailored to the target group. For nurses of the obstetrics ward, access to training on measuring neonatal vital signs should be provided. Focus must be placed on tackling reported organizational problems, including capacity shortage and suboptimal information transfer. A culture of collective responsibility of both the obstetrics and neonatology wards should be created by collective meetings and a collective point of contact. Local capacity analysis should be conducted to asses local needs and inform the team. Information transfer from the obstetrics to neonatology ward should be made as easy as possible, by using checklists, smart phrases, order sets and autofill. Integration of the EOS calculator in all Dutch EHR programs should be pursued. A smartphone application tailored to the national setting might be helpful, yet should not be obligatory. Evaluation should both measure the success of implementation and success of the innovation. It should be evaluated to what extent the expected facilitators and barriers found is this study are comparable to those after implementation, and data in the EHR should be used to analyze if implementation indeed resulted in the intended effects of the EOS calculator, such as the reduction in antibiotic prescriptions. Evaluation-based points of action should be clearly communicated with the team in a timely manner to create an environment of shared learning.

## 5. Conclusions

Our study showed a variety of barriers and facilitators of EOS calculator implementation among all relevant groups of stakeholders. Based on these findings, the EOS calculator can potentially be implemented, improving the experience of healthcare personnel as well as ultimately improving patient outcomes.

## Figures and Tables

**Table 1:** Relevant barriers and facilitators reported by all respondents.

	Discipline					Chi-Square (*p*-Value)
	PN n = 149	PO n = 84	NO n = 108	NN n = 124	Total n = 465	
n (%) Agree/Important						
Relevant Barriers						
Inner setting						
Lack of capacity on departments where neonates receive care	40 (26.8) ^a^	38 (45.2) ^b^	54 (50.0) ^b^	31 (25.0) ^a^	163 (35.1)	<0.001
Problems with handover of maternal information, from obstetric to neonatology ward	77 (52.0) ^a^	14 (16.7) ^b^	10 (9.3) ^b^	21 (16.9) ^b^	122 (26.3)	<0.001
**Relevant facilitators**						
Intervention characteristics						
Reduction of short- and long-term neonatal side effects (e.g., catheter-related infections, gastro-intestinal symptoms, altered microbiome, increased allergy risk)	148 (99.3)	82 (100)	101 (93.5)	120 (96.8)	451 (97.4)	NA
Reduction of neonatal antibiotic prescriptions	140 (94.0)	69 (83.1)	97 (90.7)	117 (94.4)	423 (91.4)	NA
Reduction of mother-child separation	131 (88.5)	78 (94.0)	105 (98.1)	118 (95.2)	432 (93.5)	NA
Reduction of blood tests in neonates at risk for infection	100 (67.1)	62 (76.5)	98 (90.7)	105 (84.7)	365 (79.0)	NA
Net shorter hospital stay of neonates at risk for infection	112 (75.2)	71 (85.5)	97 (89.8)	105 (85.4)	385 (83.2)	NA
Outer setting						
Endorsement of EOS calculator by NVK	109 (73.2)	54 (69.2)	54 (58.1)	72 (64.9)	289 (67.1)	NA
Inner setting						
Integration of EOS calculator in electronic health record	114 (76.5)	69 (84.1)	91 (85.0)	88 (73.9)	362 (79.2)	NA
Individual characteristics						
Believing the EOS calculator is safe to use	123 (82.6)	63 (75.0)	88 (81.5)	104 (83.9)	378 (81.3)	NA
Believing the EOS calculator will be effective in reducing antibiotic prescriptions	126 (84.6)	63 (75.0)	85 (78.7)	107 (86.3)	381 (81.9)	NA
Implementation process						
Providing education on the EOS calculator	109 (73.2)	61 (75.3)	99 (91.7)	111 (89.5)	380 (82.3)	NA
Providing feedback on implementation results of own department	87 (58.4)	46 (56.1)	75 (69.4)	86 (69.9)	294 (63.3)	NA

Respondents answered statements on a 5-point Likert scale. Percentages display number of respondents that indicated to agree with statement (4 = agree + 5 = totally agree) or rated the statement important (4 = important + 5 = very important). Only relevant facilitators and barriers (reported by >10% and >50% of the total group, respectively) are displayed. Background colors refer to one of the five CFIR domains, as displayed in Figure 1. EOS = early-onset sepsis; PN = physicians of the neonatology ward; PO = physicians of the obstetrics ward; NO = nurses of the obstetrics ward; NN = nurses of the neonatology ward; ‘NA’ denotes not assessed; ^a^ and ^b^ denote categories in which proportions did not significantly differ from each other after Bonferroni correction of chi-square test.

**Table 2:** Relevant barriers and facilitators reported by subgroups.

	Discipline			
	PN n = 149	PO n = 84	NO n = 108	NN n = 124
n (%) Agree/Important				
Relevant Barriers				
Individual characteristics				
Expecting more neonates to be admitted to the hospital	NA	10 (12.2)	NA	NA
Expecting increased workload	NA	29 (35.4)	NA	NA
Not thinking obstetric nurses are adequately trained to check neonatal vital signs	54 (37.8)	11 (13.4)	NA	NA
Not feeling competent to adequately measure heart rate	NA	NA	11 (11.4)	NR
Not feeling competent to adequately measure respiratory rate	NA	NA	14 (14.4)	NR
Implementation process				
Not timely being informed about changes in physicians’ protocols	NA	NA	57 (58.8)	54 (50.9)
**Relevant facilitators**				
Intervention characteristics				
Availability of an EOS calculator smartphone application	NR	NR	50 (52.1)	NR
Inner setting				
Feeling the current NVK guideline should be replaced	111 (74.5)	NR	NR	NR
Feeling that currently antibiotics are prescribed too often	113 (79.0)	NA	NA	NA
Clear communication with physicians about reasons for policy choices	NA	NA	93 (95.9)	102 (96.2)
Implementation process				
Local implementation team, as accessible point of contact	NR	NR	65 (61.9)	65 (57.0)

Respondents answered statements on a 5-point Likert scale. Percentages display number of respondents that indicated to agree with statement (4 = agree + 5 = totally agree) or rated the statement important (4 = important + 5 = very important). Only relevant facilitators (reported by >50% of subgroup) and relevant barriers (reported by >10% of subgroup) are displayed. Background colors refer to one of the five CFIR domains, as displayed in Figure 1. EOS = early-onset sepsis; PN = physicians of the neonatology ward; PO = physicians of the obstetrics ward; NO = nurses of the obstetrics ward; NN = nurses of the neonatology ward; ‘NA’ denotes not assessed: facilitator/barrier was not questioned in this group. ‘NR’ denotes not relevant: facilitator/barrier was questioned, yet not found to be relevant in this group.

**Table 3:** Relevant barriers and facilitators reported by EOS calculator users.

	Discipline			
	PN n = 149	PO n = 84	NO n = 108	NN n = 124
n (%) Agree/Important				
Relevant Barriers				
Intervention characteristics				
Encountering textual or substantive uncertainties when using the EOS calculator	5 (11.4)	NA	NA	NA
**Relevant facilitators**				
Intervention characteristics				
Care for neonates with sepsis risk is more uniform since implementation of EOS calculator	30 (68.2)	12 (75.0)	10 (66.7)	15 (60.0)
Individual characteristics				
Thinking parents of neonates agree with EOS calculator recommendations	36 (81.8)	10 (62.5)	11 (73.3)	21 (84.0)
The EOS calculator supports in making clinical decisions	39 (88.6)	NA	NA	NA

Respondents answered statements on a 5-point Likert scale. Percentages display number of respondents that indicated to agree with statement (4 = agree + 5 = totally agree). Only relevant facilitators (reported by >50% of subgroup) and relevant barriers (reported by >10% of subgroup) are displayed. Background colors refer to one of the five CFIR domains, as displayed in Figure 1. EOS = early-onset sepsis; PN = physicians of the neonatology ward; PO = physicians of the obstetrics ward; NO = nurses of the obstetrics ward; NN = nurses of the neonatology ward; ‘NA’ denotes not assessed: facilitator/barrier was not questioned in this group. ‘NR’ denotes not relevant: facilitator/barrier was questioned, yet not found to be relevant in this group.

**Table 4:** Recommendations for an implementation strategy based on survey results and CFIR-ERIC matching tool.

Identified Implementation Determinant	Recommendations
Visibility of relative advantages/fostering tension for change	Conduct local discussion meetings about EOS calculator practices Identify and prepare early adopters and local champions, who can motivate and educate colleagues Share data on implementation progress and innovation results
Stakeholder education	• Develop always accessible educational materials, such as an online instructional video • Conduct discipline-specific educational meetings ○ Nurses: practical instructions + clinical lesson ○ Physicians: practical instructions + underlying evidence
Evaluation and feedback	Develop and implement tools for quality monitoring using EHR data Collect and evaluate feedback on the implementation process per department/hospital Collect and evaluate data on intended innovation results (eg. antibiotics reduction, less blood tests) per hospital, region and nation Make evaluation data available and insightful for all stakeholders Actively communicate evaluation-based points of action, preferably directly supporting clinical practice
Integration of EOS calculator in EHR	Integrate EOS calculator in all Dutch EHR programs that are currently used Use autofill of EOS calculator fields with information available in medical record
EOS calculator smartphone application	Develop easy-to-use smartphone application, tailored to the national setting Make application available, yet not obligatory for EOS calculator use
Lack of capacity (staff and room shortage)	Conduct a capacity analysis per hospital Address the possible gap between capacity expectations actual capacity
Problems with maternal information handover	• Create culture of joint responsibility between physicians of obstetrics and neonatology ward through collective meetings and a collective point of contact • Facilitate easy information transfer, using: ○ Standardized forms, point-of-care available checklists, smart texts, order sets, autofill
Obstetric nurses not well trained to measure vital signs	Perform needs assessment per department Facilitate practical training (peer to peer) Communicate taken actions to physicians

## Data Availability

Deidentified data will be shared upon request.

## Supplementary Materials

The following supporting information can be downloaded at https://www.mdpi.com/article/10.3390/children10101682/s1. Table S1: Survey preparation: literature+ interviews; Table S2: Survey questions; Figure S1: Capacity shortage; Figure S2: Stakeholders’ educational preferences.
