# Supplementary material for: Barriers and Facilitators to the Implementation of the Early-Onset Sepsis Calculator: A Multicenter Survey Study
Source: Children (Basel). 2023 Oct 12;10(10):1682. doi: 10.3390/children10101682 (PMC10605684; doi:10.3390/children10101682)
Supplement: Supplementary file 1 [file children-10-01682-s001.zip › Nieuwe map met inhoud 2/Supplementary file 2 - Table S2.docx]

**Supplemental Table 1**

Survey questions | EOS calculator implementation

| **Background questions** | |
| --- | --- |
| *Question* | *Answer options* |
| Please indicate your discipline | - Paediatrician - Neonatologist - Neonatology fellow - Paediatric resident (not in training) - Paediatric resident (in training) - Neonatology physician assistant - Gynaecologist - Perinatologist - Gynaecology resident (not in training) - Gynaecology resident (in training) - Clinical obstetrician - Obstetric nurse - Nursing assistant - Paediatric nurse - Neonatology nurse - NICU nurse |
| Please indicate the current guideline that is used in your hospital for managing neonates at increased risk for infection | Multiple answers may be checked:   - Guideline of the Dutch National Association of Paediatrics *(Preventie en behandeling van early-onset neonatale infecties, versie juli 2017)* - EOS calculator guideline - Local guideline - Other - I do not know |
| **General questions** | |
| *Optimal guideline implementation is influenced by several factors.*    *Too what extent are the following factors important to you for working with the EOS calculator?* | |
| *Statement* | *Answer options* |
| Robust scientific evidence of effectivity | 5-point Likert scale  Not important at all – Very important |
| Decrease of negative side effects of antibiotic use in neonates |  |
| Reduction of antibiotic prescriptions |  |
| Reduction of mother-child separation in the first days of life |  |
| Net shorter hospital stay of neonates postpartum |  |
| Reduction of neonatal blood tests |  |
| Availability of an EOS calculator smartphone application |  |
| Integration of the EOS calculator in the Electronic Health Record |  |
| Endorsement of the EOS calculator by the Dutch National Association of Paediatrics |  |
| Education about the EOS calculator |  |
| A local implementation team, as point of contact for question regarding implementation |  |
| Feedback on your department’s implementation results |  |
| Insight in other hospitals’ implementation results |  |
| Which type of education would you like to receive when the EOS calculator is going to be implemented? | Multiple answers may be checked:   - Clinical lesson about early onset sepsis - Presentation about the scientific evidence underlying the EOS calculator - Presentation with practical explanation about how to use the EOS calculator, including case examples - Instructional video - I do not know |
| *Statement* | *Answer options* |
| I would like replacement/amendment of the current guideline | 5-point Likert scale  Totally disagree – Totally agree |
| I believe the EOS calculator will be effective in reducing unnecessary antibiotic exposure among neonates |  |
| I believe it is safe to use the EOS calculator |  |
| *The EOS calculator less frequently advises starting antibiotic treatment. A group of neonates is instead observed for 24 hours with frequent measurement of vital signs by the nursing staff. Depending on how care is arranged in your hospital, this may may mean that part of the care is transferred from the neonatology department to the obstetrics department.* | |
| *Question* | *Answer options* |
| Do you expect implementation of the EOS calculator will give problems in your hospital with regard to capacity? | Yes/no |
| If yes, on which department? | - NICU - High care/Post-IC - Medium Care - Obstetrics/maternity ward |
| If yes, for what reason? | - Room shortage - Staff shortage - Other |
| *Too fill in de EOS calculator, the following maternal factors are required:*   - *Gestational age (weeks + days)* - *Highest maternal antepartum temperature (Celsius)* - *Duration of rupture of membranes (hours + minutes)* - *Maternal GBS status (negative/positive/unknown)* - *Type of intrapartum antibiotics (yes/no, type, time of first gift)*   *This information should be transferred from the obstetrics ward tot neonatology ward. Indicate to what extent you agree to the following statement.* | |
| Statement/question | *Answer options* |
| I think the information transfer of maternal data will be a source of problems when using the EOS calculator | 5-point Likert scale  Totally disagree – Totally agree |
| Please explain your answer | Text box |

| **Questions for physicians of the neonatology department** | |
| --- | --- |
| *Question* | *Answer options* |
| The current Dutch National Guideline too often advises starting antibiotics for neonates at risk for early-onset sepsis | 5-point Likert scale  Totally disagree – Totally agree |
| The risk factors described in the current Dutch National Guideline lead to confusion |  |
| Care for neonates at risk for early-onset sepsis is currently sufficiently uniform |  |
| I do not feel competent to use a digital tool |  |
| I think maternity/obstetric nurses are adequately trained to measure neonatal vital signs |  |
| I think neonatology nurses are adequately trained to measure neonatal vital signs |  |
| When deciding whether or not to start antibiotic treatment in neonates, I would rather rely on my own clinical experience than on a guideline/tool |  |
| **Questions for physicians of the neonatology department – using the EOS calculator** | |
| *Statement* | *Answer options* |
| I think parents will agree with the policy based on the EOS calculator recommendations | 5-point Likert scale  Totally disagree – Totally agree |
| Care for neonates at risk for infection is more uniform since using the EOS calculator |  |
| Using the EOS calculator supports me in making the right policy choices |  |
| I am worried to miss more cases of sepsis / start antibiotics too late when using the EOS calculator |  |
| When using the EOS calculator, I encountered substantive/textual uncertainties |  |

| **Questions for physicians of the obstetrics department** | |
| --- | --- |
| *Statement* | *Answer options* |
| I do not feel competent to use a digital tool | 5-point Likert scale  Totally disagree – Totally agree |
| I think maternity/obstetric nurses are adequately trained to measure neonatal vital signs |  |
| I think that due to EOS calculator implementation more neonates from primary care will be admitted to the hospital |  |
| I think delivering information about maternal factors required for the EOS calculator will lead to increased workload compared to the current situation |  |
| **Questions for physicians of the obstetrics department – using the EOS calculator** | |
| *Statement* | *Answer options* |
| I think parents will agree with the policy based on the EOS calculator recommendations | 5-point Likert scale  Totally disagree – Totally agree |
| Care for neonates at risk for infection is more uniform since using the EOS calculator |  |
| Since using the EOS calculator, it is easier for me to inform parents when questions arise about neonatal policy |  |

| **Questions for nurses of the obstetrics/neonatology department** | |
| --- | --- |
| *Statement* | *Answer options* |
| At my department, nurses are timely being informed about changes in physicians’ protocols | 5-point Likert scale  Totally disagree – Totally agree |
| Clear communication with physicians about reasons for policy choices makes it easier for me to perform my tasks |  |
| I feel competent to adequately measure neonatal heart rate |  |
| I feel competent to adequately measure neonatal temperature |  |
| I feel competent to adequately measure neonatal respiratory rate |  |
| **Questions for nurses of the obstetrics/neonatology department – using the EOS calculator** | |
| *Statement* | *Answer options* |
| I think parents will agree with the policy based on the EOS calculator recommendations | 5-point Likert scale  Totally disagree – Totally agree |
| Care for neonates at risk for infection is more uniform since using the EOS calculator |  |
| Since using the EOS calculator, it is easier for me to inform parents when questions arise about neonatal policy |  |
