# Supplementary material for: Barriers and Facilitators to the Implementation of the Early-Onset Sepsis Calculator: A Multicenter Survey Study
Source: Children (Basel). 2023 Oct 12;10(10):1682. doi: 10.3390/children10101682 (PMC10605684; doi:10.3390/children10101682)
Supplement: Supplementary file 1 [file children-10-01682-s001.zip › Nieuwe map met inhoud 2/Supplementary file 1 - Method + Table S1.docx]

| **Consolidated Framework for Implementation Research Constructs**  Survey preparation |
| --- |
|  |

**Method**

Literature search

A Pubmed search was carried out on February 2^nd^ of 2022 to identify studies describing barriers and facilitators of EOS calculator implementation, using the following terms: (1) early onset sepsis [title and abstract] AND (2) calculator [title and abstract]. Twenty-two articles described at least one possible barrier or facilitator of EOS calculator implementation. Identified factors were allocated to one of the thirty-nine constructs of the CFIR, using the CFIR codebook. A summary of identified factors, allocated to CFIR construct, can be found in the table below.

Semi-structured interviews

In June and July of 2022, 12 stakeholder interviews were performed. Aiming to obtain divergent insights and represent all stakeholder groups, purposive sampling was used to select participants. Eligible participants were the previous mentioned stakeholders, working in a Dutch hospital at time of inclusion. For recruiting participants, physicians allied to our Pediatric Research Evaluation Network (PREN) were asked to introduce eligible candidates in their hospital. An interview guide was developed using a semi-structured format, consisting of five general questions, followed by some more in-depth questions. All questions were based on the CFIR and were formulated simple, open-ended and non-biased. Both internal testing as well as expert assessment were performed to optimize relevance, readability and order of interview questions. Twelve semi-structured interviews were performed, including two paediatricians, one neonatologist, three paediatric residents, one gynaecologic resident, one gynaecologist, one clinical obstetrician, one neonatology nurses and two maternity nurses. Interviews were executed via an online medium, lasted approximately 30 minutes and were audio-recorded after informed consent was given. Audio tapes were transcribed into a point-by-point list of reported statements about possible barriers and facilitators, which were deductively coded to one of the thirty-nine CFIR constructs, using the CFIR codebook. An overview of interview quotations, allocated to CFIR construct, can be found in the table below.

**Results**

| *Construct* | | *Literature*  *Summary of possible barriers and facilitators of the EOS calculator in literature* | | *Interviews*  *Reflective quotations per construct. Letters after quotations reflect stakeholder groups that made similar comments during the interviews. PN = physicians of neonatology ward, PO = physicians of obstetrics ward, NO = nurses of the obstetrics ward, NN = nurses of the neonatology ward* | |
| --- | --- | --- | --- | --- | --- |
|  | | **Enablers** | **Barriers** | **Enablers** | **Barriers** |
| **I. INTERVENTION CHARACTERISTICS** | | | | | |
| A | Intervention Source  *Perception of key stakeholders about whether the intervention is externally or internally developed.* |  |  |  | ‘Calculator is very ‘American’, not focused on the Dutch situation.’ (PO) |
| B | Evidence Strength & Quality  *Stakeholders’ perceptions of the quality and validity of evidence supporting the belief that the intervention will have desired outcomes.* |  |  | ‘There is trust in the underlying evidence of the EOS calculator, as we know that currently way too many antibiotics are prescribed when using the current guideline.’(PN)  ‘If it is clear that the calculator is based on high quality evidence, people are more inclined to use the calculator. But they do not know that without explanation.’ (PN, PO) |  |
| C | Relative Advantage  *Stakeholders’ perception of the advantage of implementing the intervention versus an alternative solution.* | -Cost effectiveness^1–3^  -Lower frequency of laboratory evaluation or blood cultures and shorter in-hospital stay^3–11^  -Reduction of mother-child separation ^8^ | -The EOS calculator may delay start of antibiotic treatment in cases that are initially asymptomatic and deteriorate later on, than current Dutch and NICE guidelines.^12,13^  -There is a higher amount of missed EOS cases when using the EOS calculator versus current guidelines.^14,15^ | ‘There will be less mother child separation.’ (PN, PO, NO, NN)  ‘It is an important benefit that less unnecessary antibiotics are prescribed.’ (PN, NN)  ‘Less antibiotics means less harm to the microbiome.’ (PN, NN) | ‘I am not sure what the EOS calculator will bring extra, as since introduction of the current guideline years ago, antibiotic descriptions already clearly decreased’ (PN)  ‘It is not clear to me what is the additional benefit of the EOS calculator to the current guideline’ (PN)  ‘I think the EOS calculator will lead to more work for gynaecologists, clinical obstetricians and residents’ (PO) |
| D | Adaptability  *The degree to which an intervention can be adapted, tailored, refined, or reinvented to meet local needs.* | -Risk-based GBS screening compatible with EOS calculator^16,17^ | -The calculator is not adapted to countries where no routine GBS screening is performed ^18,19^ |  | ‘Temperature is stated in Fahrenheit in the description of clinical categories, we use Celsius’ (PN)  ‘I think people usually do not know national EOS incidence, it may withhold them to fill in de calculator’ (PN, PO)  ‘GBS specific antibiotics and broad-spectrum antibiotics are the same in the Netherlands’ (PN)  ‘Apgar score of <5 is used to discriminate between clinically ill and the other categories. I think however, that this is really dependent on the situation directly after the child is born and is not always a reason for antibiotic treatment.’ (PN) |
| E | Trialability  *The ability to test the intervention on a small scale in the organization, and to be able to reverse course (undo implementation) if warranted* |  |  |  |  |
| F | Complexity  *Perceived difficulty of implementation, reflected by duration, scope, radicalness, disruptiveness, centrality, and intricacy and number of steps required to implement.* |  |  | ‘I think for nurses, using the EOS calculator may not change much in our daily work.’ (NN)  ‘There are many simple to recognize factors incorporated into the calculator.’ (PN, PO, NN) | ‘The gynaecologists have to provide us with all relevant information about the mother, if they don’t or only one thing is missing, we cannot fill in the calculator. I think that is going to be complex to arrange’ (PN)  ‘Very specific values have to be filled in the calculator, is there no way to make that easier?’ (PN, PO)  ‘I think it can cause some confusion that neonates can change from risk group after initial assessment (PN)’ |
| G | Design Quality & Packaging  *Perceived excellence in how the intervention is bundled, presented, and assembled.* |  |  | ‘It is clear what needs to be filled in.’ (PO, PN, NN, NO)  ‘The calculator is easy to fill in for everyone, very dummy proof.’ (PO, PN, NN, NO)  ‘A Dutch version of the calculator, possibly using a smartphone application or just on the website may be useful.’ (PN, PO, NO) | ‘A clear algorithm or protocol for nurses stating which vital signs they have to measure, how often and when exactly they should call the supervising physician is missing now.’ (NN, NO) |
| H | Cost  *Costs of the intervention and costs associated with implementing the intervention including investment, supply, and opportunity costs.* |  |  |  |  |
| **II. OUTER SETTING** | | | | | |
| A | Patient Needs & Resources  *The extent to which patient needs, as well as barriers and facilitators to meet those needs, are accurately known and prioritized by the organization.* |  |  | ‘I think parents will be much more relaxed when their newborn doesn’t necessarily need antibiotics, but can be observed’. (NO, NN)    ‘IV treatment and antibiotics always make parents very worried.’ (NO, NN) | ‘I think parents do not want to stay 24 hours when their newborn is doing well. Now they often stay only 12 hours.’ (PN, NN, NO, PO) |
| B | Cosmopolitanism  *The degree to which an organization is networked with other external organizations.* |  |  |  |  |
| **III. INNER SETTING** | | | | | |
| A | Structural Characteristics  *The social architecture, age, maturity, and size of an organization.* |  |  |  |  |
| B | Networks & Communications  *The nature and quality of webs of social networks and the nature and quality of formal and informal communications within an organization.* |  |  |  |  |
| C | Culture  *Norms, values, and basic assumptions of a given organization.* |  | -It may be uncommon for well babies to stay for prolonged observation at the postnatal ward^18^ |  |  |
| D | Implementation Climate  *The absorptive capacity for change, shared receptivity of involved individuals to an intervention, and the extent to which use of that intervention will be rewarded, supported, and expected within their organization.* |  |  | ‘As long policy is stated in the national guideline, our team will follow the recommendations.’ (PN) | ‘Every innovation that increases workload is hard to implement at our department, because of the high workload. It sounds like the EOS calculator will be more work.’ (PO) |
| 1 | Tension for Change  *The degree to which stakeholders perceive the current situation as intolerable or needing change.* |  |  | ‘The current guideline is not followed often, as our department thinks the guideline advises antibiotic treatment way too often, also when the neonate is totally fine.’(PN)  ‘I think a clearer guideline is needed, because there is often discussion about the current guidelines’ risk factors.’ (PO)  ‘The current guideline allows free interpretation of individual paediatricians. It is unclear to us why one neonate has to be observed for 12 hours and the other for 24 hours.’ (NO)  ‘As there is currently no uniform policy, it is hard to inform parents on what to expect. I hope use of the EOS calculator will help us.’ (PO, NO) |  |
| 2 | Compatibility  *The degree of tangible fit between meaning and values attached to the intervention by involved individuals, how those align with individuals’ own norms, values, and perceived risks and needs, and how the intervention fits with existing workflows and systems.* |  |  | ‘I think the EOS calculator fits really well in our current workflow, as not much has to be changed. The calculator consists of familiar factors. (NN, PN, PO) | ‘I think the transfer of maternal information will be problematic, as the gynaecologist is often busy after birth, but the EOS calculator needs all maternal factors filled in and those should be provided by the gynaecologist.’ (PN)  ‘It is not standard practice to note exact duration of ruptured membranes, and we often don’t know.’ (PO, PN)  ‘It is hard to receive all maternal information timely, and the filled in consultation form follows often hours after birth or is incomplete. That is also a problem with the current guideline. However, without the information, we cannot use the EOS calculator.’ (PN).  ‘After birth we have to do a lot of practical stuff, like suturing, and we do not have time to do our administration the first hours.’ (PO)  ‘The EOS calculator always advises at least 24h observation, which is not practically compatible with our agreement on daytime discharge only. That means that if we choose to apply the EOS calculator that when a baby is born in de evening, parents should stay for two nights.’ (PO, NO) |
| 3 | Relative Priority  *Individuals’ shared perception of the importance of the implementation within the organization.* |  |  | ‘I think we all agree that the current situation is not optimal, so we need new recommendations.’ (PN) |  |
| 4 | Organizational Incentives & Rewards  *Extrinsic incentives such as goal-sharing awards, performance reviews, promotions, and raises in salary, and less tangible incentives such as increased stature or respect.* |  |  |  |  |
| 5 | Goals and Feedback  *The degree to which goals are clearly communicated, acted upon, and fed back to staff, and alignment of that feedback with goals.* |  |  |  |  |
| 6 | Learning Climate  *A climate in which: a) leaders express their own fallibility and need for team members’ assistance and input; b) team members feel that they are essential, valued, and knowledgeable partners in the change process; c) individuals feel psychologically safe to try new methods; and d) there is sufficient time and space for reflective thinking and evaluation.* |  |  |  |  |
| E | Readiness for Implementation  *Tangible and immediate indicators of organizational commitment to its decision to implement an intervention.* |  |  |  |  |
| 1 | Leadership Engagement  *Commitment, involvement, and accountability of leaders and managers with the implementation.* |  |  |  |  |
| 2 | Available Resources  *The level of resources dedicated for implementation and on-going operations, including money, training, education, physical space, and time.* |  | - In some health institutes there may be a lack of available resources for enhanced monitoring and serial clinical observations^20,21^ | ‘For some innovations we have instructional videos available, I think that will really help. (NN) | ‘I think capacity will really be a problem. There is already shortage of personnel and rooms at the obstetrics department.’ (PO, PN, NN, NO)  ‘The EOS calculator will lead to increased need for capacity at the obstetrics department because:   - The standard observation period will be 24 hours, now it is often 12 hours - More neonates will stay at the obstetrics ward with their mom, since they do receive antibiotic treatment at the neonatal unit - Nurses have to do frequent measurements of neonatal vital signs, but they do not have time for that.’ (PO, NO, PN)   ‘It is always hard to educate and reach all involved people because we work in shifts.’ (NO, NN) |
| 3 | Access to Knowledge & Information  *Ease of access to digestible information and knowledge about the intervention and how to incorporate it into work tasks.* | - Guideline/tool integration into Electronic Health record ^22^ |  | ‘It is easy to find the calculator online.’ (PO, PN)  ‘Integration of the EOS calculator in the Electronic Health Record would be really helpful, I think necessary actually.’ (PO, PN, NN, NO)  ‘A smartphone application would be useful when you are not near a desktop.’ (PO, PN)  ‘The EOS calculator should not be only accessible through a smartphone app, but also through a link in the protocol.’ (PN) |  |
| **IV. INDIVIDUAL CHARACTERISTICS** | | | | | |
| A | Knowledge & Beliefs about the Intervention  *Individuals’ attitudes toward and value placed on the intervention as well as familiarity with facts, truths, and principles related to the intervention.* |  |  | ‘I think the calculator will lead to more uniform care.’ (PO, PN, NN, NO)  ‘I expect there will be more consistency between obstetrics and neonatology.’ (G)  ‘The EOS calculator will support me in making the right choice when in doubt.’ (PN)  ‘I think it will really help young doctors to have such a clear tool to determine policy, especially during night shifts.’ (PN)  ‘When using the EOS calculator, I will find it easier to explain to parents and colleagues why I do or do not start with antibiotic treatment.’ (PN) | ‘I am worried that doctors stop thinking themselves about reasons for choosing antibiotic treatment, is filling in the calculator is so easy. It is important that we keep understanding why we make certain choices.’ (PN, NN)  ‘I am afraid that we will start antibiotics too late. The EOS calculator advises to wait 2-4 hours in the equivocal group, depending on the symptoms. But sometimes you just want to start. I think in practice people do not dare to wait, especially when there is instability of temperature or a combination of symptoms.’ (PN)  ‘I do not know where the calculator algorithm is based on. I need this knowledge to use it properly.’ (PO, PN)  ‘In my opinion, neonates with just one risk factor can often be discharged after 12 hours of observation, 24 hours is too long.’ (PN). |
| B | Self-efficacy  *Individual belief in their own capabilities to execute courses of action to achieve implementation goals* |  |  |  | ‘I know that many nurses at my department do not really feel competent to measure neonatal vital signs, especially not the heart and respiratory rate.’ (NO) |
| C | Individual Stage of Change  *Characterization of the phase an individual is in, as he or she progresses toward skilled, enthusiastic, and sustained use of the intervention* |  |  |  | ‘I know that strength of habit really takes over soon in our team, so it should be really clear why a new intervention is so important.’ (PO)  ‘People will keep referring to the old protocol and make choices based on that.’ (G, PN) |
| D | Individual Identification with Organization  *A broad construct related to how individuals perceive the organization, and their relationship and degree of commitment with that organization.* |  |  |  |  |
| E | Other Personal Attributes  *A broad construct to include other personal traits such as tolerance of ambiguity, intellectual ability, motivation, values, competence, capacity, and learning style.* |  |  | ‘Nurses really appreciate it when things are going according to protocol, which seems to be easier with the EOS calculator.’ (NN) | ‘I find it hard that neonates I would otherwise have prescribed antibiotics are now observed, and that the observation is done by nurses who are not really well trained in measuring neonatal vitals.’ (PN)  ‘Obstetric nurses are only educated to care for mothers and healthy neonates.’ (PN, NO) |
| A | Planning  *The degree to which a scheme or method of behavior and tasks for implementing an intervention are developed in advance, and the quality of those schemes or methods.* |  |  |  |  |
| B | Engaging  *Attracting and involving appropriate individuals in the implementation and use of the intervention through a combined strategy of social marketing, education, role modeling, training, and other similar activities.* | - |  | ‘To activate people for change, there should be a clear message or goal accompanying the change.’ (PO, PN, NN, NO)  ‘I think people will be really motivated about the fact that there is less mother child-separation. That should be emphasized.’ (NO, PO)  ‘We need education on the EOS calculator, about the why, when and what.’ (PN, PO, NN, NO)  ‘Promotion of the EOS calculator can be started by giving motivational and explanatory presentations.’ (NN, NO)  ‘Education about the evidence base is important: how are the risk factors determined, how much reduction of antibiotic treatment is expected, how often are cases missed?’ (PN)  ‘There should be training, really focused on daily practice: explanation on how to fill in the calculator and practicing with case examples.’ (PO, NO, NN, PN)  ‘We need frequent reminders that the EOS calculator is the new clinical practice, and we should work accordingly.’ (PO) | Nurses often do not know about changes in physicians’ protocols, which makes it harder to follow policy. We need timely information. (NN, NO) |
| 1 | Opinion Leaders  *Individuals in an organization who have formal or informal influence on the attitudes and beliefs of their colleagues with respect to implementing the intervention.* |  |  |  |  |
| 2 | Formally Appointed Internal Implementation Leaders  *Individuals from within the organization who have been formally appointed with responsibility for implementing an intervention as coordinator, project manager, team leader, or other similar role.* |  |  | ‘There should be a local implementation team that is always available when questions arise.’ (NO, NN, PO)  I think someone in the team should be the leader of the implementation, and that person should be really visible.’ (NN) |  |
| 3 | Champions  *“Individuals who dedicate themselves to supporting, marketing, and ‘driving through’ an [implementation]” [101] (p. 182), overcoming indifference or resistance that the intervention may provoke in an organization.* |  |  | ‘We need someone who takes the lead locally and is really motivated to make in work.’ (PN, NN, NO) |  |
| 4 | *Individuals who are affiliated with an outside entity who formally influence or facilitate intervention decisions in a desirable direction.* |  |  |  |  |
| C | Executing  *Carrying out or accomplishing the implementation according to plan.* |  |  | ‘During implementation there should be clear agreements on where the neonate will be observed: at the neonatology ward or the maternity ward?’ (NN, NO) | ‘Often when something new is implemented, the old protocol is still in the document list of our department, which is really confusing.’(PN, PO) |
| D | Reflecting & Evaluating  *Quantitative and qualitative feedback about the progress and quality of implementation accompanied with regular personal and team debriefing about progress and experience.* |  |  | ‘It is important that everyone gets the chance to speak up about things that are not going well, also after implementation’ (PN, NN)  ‘After implementing the calculator, it should be evaluated what the effect is on neonatal care, not only at the department level but also cross-hospital.’ (PN)  ‘It should be monitored within the department why paediatricians still choose to deviate from the EOS calculator recommendations.’ (PN)  ‘Motivation is increased when you get insight in the positive effects of a new innovation at your department, so this should be evaluated and given as feedback to the team.’ (PN, PO, NN, NO) | 24-27 |

1. Gong CL, Dasgupta-Tsinikas S, Zangwill KM, Bolaris M, Hay JW. Early onset sepsis calculator-based management of newborns exposed to maternal intrapartum fever: a cost benefit analysis. *J Perinatol*. 2019;39(4):571-580. doi:10.1038/S41372-019-0316-Y

2. Cussen A, Guinness L. Cost savings from use of a neonatal sepsis calculator in Australia: A modelled economic analysis. *J Paediatr Child Health*. 2021;57(7):1037-1043. doi:10.1111/JPC.15384

3. Achten NB, Visser DH, Tromp E, Groot W, van Goudoever JB, Plötz FB. Early onset sepsis calculator implementation is associated with reduced healthcare utilization and financial costs in late preterm and term newborns. *Eur J Pediatr*. 2020;179(5):727-734. doi:10.1007/S00431-019-03510-9

4. Zayek M, Bhat J, Bonner K, et al. Implementation of a Modified Neonatal Early-onset Sepsis Calculator in Well-baby Nursery: a Quality Improvement Study. *Pediatr Qual Saf*. 2020;5(4):e330. doi:10.1097/PQ9.0000000000000330

5. Fischer A, Mowrer MC, Shallat S, Walker L, Shallat J. Ensuring a Locally Tailored Response to Early Onset Sepsis Screening Meets or Exceeds the Performance of Published Approaches. *Hosp Pediatr*. 2020;10(10):877-883. doi:10.1542/HPEDS.2020-0153

6. Loughlin LM, Knowles S, Twomey A, Murphy JFA. The Neonatal Early Onset Sepsis Calculator; in Clinical Practice. *Ir Med J*. 2020;113(4).

7. Leonardi BM, Binder M, Griswold KJ, Yalcinkaya GF, Walsh MC. Utilization of a Neonatal Early-Onset Sepsis Calculator to Guide Initial Newborn Management. *Pediatr Qual Saf*. 2019;4(5):e214. doi:10.1097/PQ9.0000000000000214

8. Bridges M, Pesek E, McRae M, Chabra S. Use of an Early Onset-Sepsis Calculator to Decrease Unnecessary NICU Admissions and Increase Exclusive Breastfeeding. *J Obstet Gynecol Neonatal Nurs*. 2019;48(3):372-382. doi:10.1016/J.JOGN.2019.01.009

9. Helmbrecht AR, Marfurt S, Chaaban H. Systematic Review of the Effectiveness of the Neonatal Early-Onset Sepsis Calculator. *J Perinat Neonatal Nurs*. 2019;33(1):82-88. doi:10.1097/JPN.0000000000000360

10. Strunk T, Buchiboyina A, Sharp M, Nathan E, Doherty D, Patole S. Implementation of the Neonatal Sepsis Calculator in an Australian Tertiary Perinatal Centre. *Neonatology*. 2018;113(4):379-382. doi:10.1159/000487298

11. Beavers JB, Bai S, Perry J, Simpson J, Peeples S. Implementation and Evaluation of the Early-Onset Sepsis Risk Calculator in a High-Risk University Nursery. *Clin Pediatr (Phila)*. 2018;57(9):1080-1085. doi:10.1177/0009922817751337

12. Kopec G, Collin M, Das A. Application of Kaiser Sepsis Calculator in culture-positive infants with early onset sepsis. *World J Pediatr*. 2021;17(4):429-433. doi:10.1007/S12519-021-00446-9

13. Snoek L, van Kassel MN, Krommenhoek JF, et al. Neonatal early-onset infections: Comparing the sensitivity of the neonatal early-onset sepsis calculator to the Dutch and the updated NICE guidelines in an observational cohort of culture-positive cases. *EClinicalMedicine*. 2022;44:101270. doi:10.1016/J.ECLINM.2021.101270/ATTACHMENT/ED894DCF-E615-40F2-9734-5DEA8C271894/MMC3.DOCX

14. pettScott PA, Lai M, Inglis GDT, Davies MW. Neonatal early-onset sepsis calculator safety in an Australian tertiary perinatal centre. *J Paediatr Child Health*. 2022;58(5). doi:10.1111/JPC.15860

15. Pettinger KJ, Mayers K, McKechnie L, Phillips B. Sensitivity of the Kaiser Permanente early-onset sepsis calculator: A systematic review and meta-analysis. *EClinicalMedicine*. 2019;19. doi:10.1016/J.ECLINM.2019.11.020

16. Kim MJ. Utility of neonatal early-onset sepsis calculator in risk-based group B Streptococcus screening approach. *Clin Exp Pediatr*. 2020;63(10):393. doi:10.3345/CEP.2020.00500

17. Achten NB, Dorigo-Zetsma JW, van Rossum AMC, Oostenbrink R, Plötz FB. Risk-based maternal group B Streptococcus screening strategy is compatible with the implementation of neonatal early-onset sepsis calculator. *Clin Exp Pediatr*. 2020;63(10):406-410. doi:10.3345/cep.2020.00094

18. Saw C, Kulasekaran K, Fernando DT, et al. Retrospective cohort study of neonatal early onset of sepsis and the role of the EOS calculator in a level II nursery. *Pediatr Neonatol*. 2021;62(5):512-521. doi:10.1016/j.pedneo.2021.05.005

19. Riskin A, Bryskin S, Zaitoon H, et al. Evaluation of Implementation of Early-Onset Sepsis Calculator in Newborns in Israel. *J Pediatr*. 2021;234:71-76.e2. doi:10.1016/J.JPEDS.2021.04.007

20. Kimpton JA, Verma A, Thakkar D, et al. Comparison of NICE Guideline CG149 and the Sepsis Risk Calculator for the Management of Early-Onset Sepsis on the Postnatal Ward. *Neonatology*. 2021;118(5):562-568. doi:10.1159/000518059

21. Cavazos R, Patil M, Gautham KS. A sepsis risk calculator can decrease antibiotic exposure in neonatal early-onset sepsis screening. *Acta Paediatr*. 2020;109(10):2166-2167. doi:10.1111/APA.15332

22. Stipelman CH, Smith ER, Diaz-Ochu M, et al. Early-Onset Sepsis Risk Calculator Integration Into an Electronic Health Record in the Nursery. *Pediatrics*. 2019;144(2). doi:10.1542/PEDS.2018-3464
