# Supplementary material for: Barriers and Facilitators to the Implementation of the Early-Onset Sepsis Calculator: A Multicenter Survey Study
Source: Children (Basel). 2023 Oct 12;10(10):1682. doi: 10.3390/children10101682 (PMC10605684; doi:10.3390/children10101682)
Supplement: Supplementary file 1 [file children-10-01682-s001.zip › Nieuwe map met inhoud 2/Supplementary file 3 - Figure F1.docx]

**Stakeholders’ expectations regarding capacity shortage**

**A.** Departments where capacity problems are expected

**B.** Reasons for expected capacity problems

*Respondents were asked to indicate on which department and for what reason they expected capacity problems. Multiple answers could be selected.*

*NICU = neonatal intensive care unit*
