# Supplementary material for: Barriers and Facilitators to the Implementation of the Early-Onset Sepsis Calculator: A Multicenter Survey Study
Source: Children (Basel). 2023 Oct 12;10(10):1682. doi: 10.3390/children10101682 (PMC10605684; doi:10.3390/children10101682)
Supplement: Supplementary file 1 [file children-10-01682-s001.zip › Nieuwe map met inhoud 2/Supplementary file 4 - Figure F2.docx]

Stakeholders’ educational preferences

*Respondents were asked to indicate which type of education they would like to receive. Multiple answers could be selected.*

*PN = physicians of neonatology ward, PO = physicians of obstetrics ward, NO = nurses of the obstetrics ward, NN = nurses of the neonatology ward*
